# Comparison of traditional and ecological dynamical training approaches in Brazilian Jiu-Jitsu: effects on Force Development, reactivity, and distress perception

**DOI:** 10.3389/fpsyg.2026.1794709

**Published:** 2026-03-20

**Authors:** Giuseppe Giardullo, Rosario Ceruso, Giuseppe Di Lascio, Manuele Taleb, Giovanni Esposito, Francesca D'Elia

**Affiliations:** 1 Research Center of Physical Education and Exercise, University of Pegaso, Naples, Italy; 2 Department of Neuroscience, Biomedicine and Movement, University of Verona, Verona, Italy; 3 Department of Human, Philosophical and Education Sciences, University of Salerno, Salerno, Italy

**Keywords:** adaptation, combat sports, martial arts, psychophysical, versatility

## Abstract

Brazilian Jiu Jitsu is a combat sport discipline characterized by high situational complexity. No studies in the literature provide a systematic scientific foundation for the comparison between traditional teaching methodologies and the ecological–dynamical approach. The aim of this study is to compare the effectiveness of two training approaches on the following parameters: Rate of Force Development, muscular reactivity, and perception of psychophysical distress. Sixty amateur and semi-professional athletes were equally divided into an experimental group and a control group. For the pre- and post-intervention assessments, the following tests were used: the Isometric Mid-Thigh Pull with a force platform for RFD; the Blaze Pod Reaction Speed test with Blaze Pods for reactivity; and *ad hoc* questionnaires to collect data on distress perception. The mixed 2 × 2 ANOVA showed that peak RFD increased by 15% in the experimental group compared with 5% in the control group (*p* = 0.020). Reaction times, also analyzed through mixed ANOVA, decreased by 15% in the experimental group compared with 7% in the control group (*p* = 0.007). Only the experimental group showed a significant reduction in perceived distress (Wilcoxon: *Z* = −4.10, *p* < 0.001). The post-intervention comparison between groups using the Mann–Whitney test revealed a significant difference in favor of the experimental group (*U* = 320.0, *p* =0.004). The results indicate that an approach based on the ecological–dynamical framework leads to greater improvements in explosive capacity, reactivity, and distress management in Brazilian Jiu Jitsu athletes compared with the traditional method.

## 1 Introduction

Brazilian Jiu Jitsu (BJJ) is a martial and sporting discipline grounded in the art of controlling and submitting an opponent through grips, joint locks, and continuous transitions (Kedziorek et al., 2025). This feature distinguishes it from striking martial arts, placing the emphasis on close-range, tactical, and constantly evolving exchanges (Santos et al., 2023). In BJJ, the athlete is immersed in a highly dynamic environment, where every motor decision arises from direct interaction with the opponent's body and with the conditions of the moment. This results in a context of great perceptual and motor complexity, requiring adaptability, proprioceptive sensitivity, and a high level of decision-making readiness (Fagundes et al., 2024). Owing to these characteristics, Brazilian Jiu Jitsu is often described as “modern chess”, in reference to its strategic complexity and the continual need to anticipate and respond to the opponent's moves, features that indeed bring it close to the game of chess (Báez-San Martín et al., 2025). From a physiological perspective, BJJ is characterized by an intermittent pattern, alternating explosive, high-intensity phases, such as attempts at throws or guard passes, with more static and isometric control phases (Esposito et al., 2025a,b,c). This alternation requires careful management of energy resources and considerable neuromuscular efficiency (Aliberti et al., 2021). According to Andreato et al. (2016), the performance profile of a BJJ athlete is defined by the integration of strength, power, endurance, and postural control, elements that together contribute to determining performance effectiveness (Andreato et al., 2016). Among the decisive capacities, the rapidity with which an athlete is able to generate force, namely the Rate of Force Development (RFD), plays a central role (Esposito et al., 2024a,b). It influences the ability to react promptly to the opponent's actions, to reverse a position, or to complete a submission (Wasacz et al., 2023). Studies by Aagaard et al. (2002) and Maffiuletti et al. (2016) have shown that training aimed at increasing neuromuscular power induces both central adaptations, linked to motor activation, and peripheral ones, with positive effects on the speed of force production. Similarly, visuomotor reactivity, understood as the ability to respond rapidly to visual or proprioceptive stimuli, represents an essential aspect of performance in BJJ, as it allows the athlete to anticipate, compensate for, or exploit sudden changes in rhythm and direction during combat (Polechoński and Langer, 2022). Alongside these physical demands, BJJ also entails a considerable psychological and physiological load, as the athlete is constantly exposed to prolonged motor and cognitive stress situations that require effective emotional regulation and the ability to manage fatigue (Raiola et al., 2025). Slimani et al. (2017) emphasized that subjective perception of effort (RPE) and psychophysical distress are useful indicators for understanding the body's overall response to training or competition, although they remain insufficiently explored in the context of BJJ. Traditionally, preparation in this discipline has been based on a prescriptive and linear approach centered on the repetition of standardized techniques isolated from the real combat context. This method has certainly contributed to consolidating technical mastery, but it tends to reduce the athlete's exposure to the variability and unpredictability that characterize actual competition. In recent years, however, interest within the coaching world of combat sports has progressively shifted toward the ecological–dynamical approach, a paradigm that has also generated significant debate within the scientific community of sport and exercise science (Esposito et al., 2024a,b). This theoretical framework interprets motor learning as the result of the continuous interaction between athlete, task, and environment, emphasizing the adaptive and situational nature of motor behavior (Davids et al., 2003; Renshaw and Chow, 2019). This model, grounded in the principles of the constraints-led approach and of the perception–action relationship, places at its center the concept of self-organization of motor behavior (Taleb et al., 2021). From this perspective, the athlete does not imitate a predefined movement pattern but constructs effective solutions by adapting to the conditions of the context (Raiola and Altavilla, 2020). Variability is therefore not an error to be corrected, but rather a functional element that stimulates flexibility and adaptability of the motor system (Esposito et al., 2025a,b,c). In BJJ, this view appears particularly relevant. The practice environment, rich in sensory stimuli and real-time decisions, naturally lends itself to training that promotes the exploration of different motor solutions and the management of situational constraints (Ceruso et al., 2020). Applying an ecological–dynamical paradigm to BJJ training could foster more authentic learning, grounded in the actual conditions of combat, capable of improving not only reactivity and neuromuscular efficiency but also the ability to regulate the psychophysical distress deriving from competition (Krabben et al., 2019). Despite the growing interest in this approach, scientific literature remains limited. Most studies on BJJ continue to focus on traditional training models and basic physiological variables, overlooking the impact of the ecological–dynamical approach on the development of skills that are fundamental for performance. To date, indeed, no solid scientific evidence exists demonstrating the effectiveness of this approach in improving key parameters such as Rate of Force Development, visuomotor reactivity, and the management of psychological distress, leaving open a central question for the optimization of practice in Brazilian Jiu Jitsu. The study aims to compare an ecological dynamical training protocol with a traditional one in Brazilian Jiu Jitsu, assessing their effects on RFD, visuomotor reactivity, and psychophysical distress, in order to identify which methodology promotes more functional adaptations for performance.

## 2 Methods

### 2.1 Design and participants

A total of 60 Brazilian Jiu Jitsu (BJJ) athletes were involved in the study and randomly and equally assigned to two working groups: an experimental group, which followed a training program based on the ecological–dynamical approach, and a control group, composed of athletes who instead continued to use a traditional preparation method. To ensure a balanced distribution between the two groups, the randomization procedure was stratified by sex, so that each group included 21 male participants and 9 female participants. The subjects were recruited from two local BJJ academies operating within the same sports facility, through direct contact with the coaches and a detailed presentation of the research project, resulting in a convenience sample composed of athletes who were available and easily accessible within the sporting environments involved. The recruitment of participants within a single training context was chosen to ensure homogeneity of practice conditions, workload, and coaching style, elements that are particularly relevant in combat sports. This approach made it possible to reduce potential confounding factors related to variability in training programmes, thereby strengthening experimental control and the internal validity of the study (Garcia-Carrillo et al., 2025). All participants took part voluntarily after receiving full information regarding the aims and procedures of the study. Specific inclusion and exclusion criteria were established for the selection of the sample, with the aim of ensuring homogeneity among participants and consistency with the objectives of the study. Athletes included in the study were required to be between 18 and 40 years of age, to have at least 12 months of continuous practice in the discipline, and to have trained no fewer than 2 times per week over the past 6 months. All subjects had to possess a valid sports medical clearance and belong to a technical level ranging from advanced white belt to brown belt, in order to represent practitioners with a consolidated degree of competence while not yet being at a professional level. Only athletes in good general health, free from recent musculoskeletal injuries or conditions that might compromise the performance of the planned physical tests, were admitted. Furthermore, to be included, participants had to declare their availability to take part in the entire experimental period and to undergo both pre- and post-intervention assessments. Participation was voluntary, and all athletes signed a written informed consent after receiving a complete explanation of the study's aims and procedures. Athletes presenting relevant cardiovascular, respiratory, neurological, or metabolic conditions were excluded, as were those who had undergone surgery in the previous 6 months or who had sustained acute injuries or chronic pain in the 3 months before the study. Participants using medications or substances capable of altering neuromuscular response or psychological state were also excluded unless their condition had been stable for at least 3 months. To ensure the quality of the collected data, athletes who did not adhere to standardized pre-test conditions were not admitted, conditions such as refraining from intense training in the 24 h preceding the assessments, from caffeine and alcohol in the 12 h before testing and ensuring adequate sleep (at least 7 h) the night before the test. Participation was also excluded for pregnant women and for athletes who could not guarantee a minimum attendance of 85% of the scheduled training sessions or who were unable to comply with the assessment timetable. All eligibility criteria were verified through a structured anamnesis interview and the completion of an individual information form, conducted by the research team prior to the randomization of participants into the experimental and control groups. The study adopted a controlled randomized experimental design with pre- and post-intervention measures, aimed at comparing the effects of two different training methodologies, one based on the ecological–dynamical approach and the other on a traditional prescriptive method, in order to assess the variables of Rate of Force Development (RFD), visuomotor reactivity, and psychological distress in BJJ athletes. Following the initial assessment phase, the 60 eligible participants were randomly assigned to one of the two groups:

- Experimental Group (EG), subjected to a training program designed according to the principles of the ecological–dynamical approach.
- Control Group (CG), which followed a program based on traditional technical and conditioning training methods.

Both groups completed 4 weekly sessions lasting approximately 90 min each, over a total period of 8 weeks. Training sessions were conducted in the same sports facility for both groups, on a standard tatami surface, using exclusively the athletes' bodies and partner interaction as the primary training tools. Exercises involved paired athletes with systematic partner rotation to maintain controlled interpersonal variability. All sessions were supervised by qualified instructors with several years of experience in Brazilian Jiu-Jitsu. The training intervention was designed according to the principles of ecological dynamics, conceptualizing motor learning as the result of continuous interaction between the athlete, the task, and the environment (Pinder et al., 2011). From this perspective, tasks were structured through the systematic manipulation of spatial, temporal, informational, and interpersonal constraints in order to promote motor exploration, functional adaptation, and decision-making under conditions representative of the Brazilian Jiu-Jitsu competitive context. The intervention description was organized in line with transparency and replicability criteria commonly adopted in experimental studies of training interventions, including task objectives, execution modalities, progression, and the role of instructors (Renshaw et al., 2022). Each session included exercises of varying complexity, in which the technical and tactical objective was achieved through realistic grappling situations and constantly evolving scenarios. The athletes were encouraged to explore different motor solutions and to make decisions based on the perceptual information provided by the partner and the context. A typical task during the initial weeks consisted of micro-sparring from the closed guard position within a restricted space, with the partner initially adopting a passive role. The objective was to promote exploration of transitions and perception of bodily pressures, while the spatial constraint increased the informational density of the task. In subsequent phases, athletes performed escapes from disadvantageous positions under time constraints (15–20 s), with rapid role alternation and progressively more active partner opposition. In this case, temporal and interpersonal constraints were manipulated to increase decision-making demands and the management of situational stress. In the final weeks, ecological sparring included simulations of real combat with variable tactical constraints, such as limitations on the use of specific limbs or mandatory transitions within defined time frames, with the aim of consolidating functional adaptation and transferring skills to the competitive context. The training program activities are presented in Table 1.

**Table 1:** Training protocol of the experimental group.

**Week**	**Main focus**	**Ecological–dynamical activities**	**Constraints/environmental manipulations**	**Adaptive goal**
1	Perceptual orientation and adaptation	Simple situational drills; free exploration of movements from static positions; interaction with a partner at controlled intensity	Spatial constraint (reduced area); unlimited time; passive partner	Promote motor exploration and perceptual awareness
2	Perception–action and tactile sensitivity	Reaction exercises to tactile and visual stimuli; grip variations; conditioned low-time attacks.	Limitation of the use of one limb; sudden task changes; semi-active partner.	Develop the ability to adapt to the partner's sensory stimuli
3	Motor adaptation and constraint variation	Micro-sparring with predefined scenarios (closed guard, half guard); exploration of multiple solutions	Reduced action time (15–20 s); role changes every round	Promote tactical adaptation and decision-making speed
4	Pressure management and reaction timing	Exit drills from disadvantageous positions; unpredictable sequences; micro-sparring with rapid attacks	Time constraints; partner with alternating roles; limited feedback	Improve motor stress management and reactivity
5	Decision-making under complex constraints	Multiple-choice scenarios (various possible submissions or transitions); delayed feedback	Random stimuli (visual or tactile); alternating tempo between rounds	Train the decision-making process in uncertain contexts
6	Dynamic transitions and adaptive coordination	Continuous transition drills between positions; fluid and adaptive exchanges between partners	Reduced combat area; increasing intensity; partial visual limitation (blindfolded)	Enhance intersegmental coordination and spatial sensitivity
7	Functional variability and self-organization	Exercises with multiple constraints; role alternation in unpredictable situations; free micro-sparring	Continuous modification of time, space, and intensity; open feedback	Foster the discovery of individual motor solutions.
8	Ecological consolidation and transfer	Ecological sparring: realistic combat simulation with tactical constraints; guided final reflection	Variable complex constraints; post-session metacognitive feedback	Consolidate adaptive learning and autonomous regulation capacity

The coaching adopted an indirect style, aimed at stimulating reflection and adaptation rather than providing prescriptive instructions. The CG followed a training program structured according to a traditional approach, based on prescriptive teaching methods. Each session was organized into three distinct phases. The first phase included a general and specific warm-up lasting 10–15 min, with joint mobility exercises, movement drills, and technical drills performed at low intensity. Subsequently, the central phase of the training (approximately 40–50 min) was dedicated to the study of techniques selected by the coach, such as throws, guard passes, transitions, or submissions, repeated in an analytical form and with a high degree of cooperation from the partner. A typical task consisted of the analytical repetition of a specific technique, such as guard passes or transitions to side control, performed in predefined sequences with a cooperative partner and minimal resistance. The session concluded with an application phase of 20–30 min, consisting of conditioned drilling exercises and rounds of free sparring, organized with uniform times and modalities for all practitioners. The coach's intervention was mainly directive: he demonstrated the technical model to be learned, and participants were instructed to reproduce it by adhering precisely to the demonstrated sequence. The feedback provided was mostly corrective and oriented toward the formal quality of the movement. The variability of the grappling situations remained limited, as the exercises were performed starting from predefined positions and with fixed roles (attacker and defender), without systematically modifying the spatial or temporal constraints of the practice. The final sparring naturally introduced a greater margin of unpredictability, but it did not involve intentional manipulations of task or environmental constraints, taking place according to the usual modalities of traditional Brazilian Jiu Jitsu training. The training activities planned for the CG are shown in Table 2.

**Table 2:** Training protocol of the control group.

**Week**	**Main focus**	**Traditional activities**	**Session structure**	**Instructional objective**
1	Initial adaptation and technical review	Low-intensity technical drills; analytical exercises on fundamental positions	Warm-up; demonstrated technique; prescribed repetitions	Reinforce fundamentals and execution precision
2	Basic techniques in cooperation	Guided repetitions of throws and guard passes with a collaborative partner	Fixed sequence of techniques; cooperative drilling	Improve fluidity in standard movements
3	Controlled application of techniques	Conditioned drills with predefined roles; moderate-resistance exercises	Technique/conditioned exercises/light sparring	Apply familiar techniques in low-variability contexts
4	Preset technical sequences	Repetition of technical combinations established by the coach	Sequence study/analytical practice/randori	Memorize technical sequences and improve coordination
5	Controlled transitions	Transition drills performed with a collaborative partner	Structured repetitions of transitions; low variability	Make standard transitions more fluid
6	Technical refinement	Focus on technical details; individual corrections from the coach	Demonstrated technique, repetitions, conditioned exercises	Increase precision and correct recurring errors
7	Consolidation of learned techniques	General review; collaborative drills; moderate-intensity randori	Consistent structure: warm-up/technique/drills/randori	Stabilize the technical skills acquired
8	Final application	Regular free sparring; final review of key techniques	Standard traditional training sessions	Verify technical execution in unconditioned sparring

In the experimental group, training load progression was achieved primarily through the gradual increase in task complexity, situational unpredictability, and decision-making demands, rather than through linear increases in physical volume. Specifically, available action time was progressively reduced, partner opposition was increased, and multiple constraint combinations were introduced. In the control group, progression predominantly involved increasing the volume of technical repetitions and the duration of sparring phases, while keeping task structure and variability levels unchanged. In both groups, tasks were only minimally adapted in response to athletes' performance, mainly to ensure safety and correct execution, without altering the overall protocol logic. Session attendance was monitored by researchers and instructors, with a minimum participation rate of 85% required for inclusion in the final analysis. All sessions were conducted according to the planned protocols, with no substantial deviations from the intended content. The presence of the same instructors throughout the intervention ensured consistency in the implementation of the training programmes across both groups. To analyze the effects of the training program, all participants underwent a series of assessments carried out both before and after the intervention period. The test battery was designed to detect neuromuscular changes, variations in response reactivity to visual stimuli, and possible modifications in the individual perception of distress. The neuromuscular component was investigated through the Isometric Mid-Thigh Pull (IMTP) in a standing position, performed using Deltas force and balance platforms connected via Bluetooth to the dedicated software. The platform operated at a sampling frequency of 1,500 Hz, allowing for a detailed analysis of the force–time curves. From each trial, indices related to the Rate of Force Development (RFD) were obtained, calculated over the temporal windows commonly used in the literature (0–50 ms, 0–100 ms, 0–200 ms) and on the peak value. Positioning required a slight flexion of hips and knees, with the trunk kept stable and the bar aligned approximately with the mid-third of the femur. Each subject performed three attempts, spaced by 2 min of recovery, and the best trial was selected for analysis, provided it showed a clean force curve free from artifacts. Visuomotor reactivity was instead measured through the Blaze Pod Reaction Speed Test, using the Blaze Pod system in a controlled mode and adapting its execution to the needs of Brazilian Jiu Jitsu. The test was performed standing, with the athletes placed in a guard stance similar to the posture assumed during waiting phases or during the reading of the opponent in upright grappling situations. In front of them, three Blaze Pods were placed on a semicircle at a distance of approximately 60–70 cm and at hip height: a configuration chosen to recall distances and intervention angles frequently found in grip attempts, hand fighting, and rapid directional changes typical of the discipline. Once the test started, one of the pods lit up according to a random sequence generated by the software, and the athlete had to quickly identify the stimulus and reach it with the hand, alternating between dominant and non-dominant, as rapidly as possible. After touching the pod, the participant returned immediately to the initial position, ready to respond to the next signal. Before beginning, athletes were instructed to maintain a minimum level of pre-activation, with hips and knees slightly flexed, so as to avoid preparatory movements that could artificially influence reaction times. Each athlete completed three 15-s attempts, during which the system automatically recorded the reaction time to each individual light stimulus. In addition to single values, the software also provided average time, minimum and maximum time, and intra-trial variability, enabling a more refined interpretation of response quality. For analysis, the mean value of the three attempts was considered, following a common procedure in studies employing visual reaction measures. This testing modality offers a realistic representation of the perceptual and motor demands of Brazilian Jiu Jitsu, as the type of stimulus, the distance, and the starting posture reproduce situations in which rapid perception and immediate action are decisive: from initial grip engagement to the defense against sudden attacks, to level changes and micro-adjustments required during stand-up exchanges. In parallel, the perception of distress was assessed through *ad hoc* questionnaires, administered at the same time points as the physical assessments. In the present study, distress was conceptualized as a negative psychological response to the situational demands of training, characterized by perceptions of mental pressure, difficulties in emotional regulation, interference of daily stress with practice quality, and a sense of psychological overload during demanding tasks (Nyhus Hagum et al., 2022). This construct is distinct from perceived exertion (RPE), which primarily reflects the physical intensity of exercise, from performance anxiety, understood as an anticipatory emotional state related to outcome evaluation, and from mental fatigue, referring to the depletion of cognitive resources resulting from prolonged or monotonous tasks. The development of the questionnaire was informed by previous context-specific approaches used in sport to assess psychophysiological perceptions related to disciplinary demands (Mao, 2025). The choice of a contextual instrument was motivated by the situational nature of Brazilian Jiu-Jitsu, characterized by prolonged physical contact, high environmental uncertainty, and continuous real-time decision-making demands, elements that are not fully captured by general psychometric scales. The questionnaire was designed to investigate key dimensions of situational distress, including mental pressure during training, post-exertion cognitive fatigue, interference of daily stress with practice quality, motivational fluctuations, and difficulties in emotional regulation in grappling situations perceived as demanding. Prior to its use in the main study, the internal consistency of the instrument was assessed using Cronbach's coefficient, which indicated good overall reliability (α = 0.81), suggesting coherent measurement of the perceived distress construct. Although the questionnaire demonstrated good internal reliability, it has not yet undergone formal external psychometric validation procedures. This limitation restricts direct comparability with standardized instruments commonly used in sport psychology; however, it was considered acceptable within the present exploratory context aimed at capturing discipline-specific forms of distress. The compilation procedure was standardized to ensure uniformity and promote data comparability. Table 3 presents the administered questionnaire.

**Table 3:** Questionnaire on distress perception in BJJ athletes.

**Question**	**Responses**
1–During BJJ training, have you felt mentally pressured or found it difficult to concentrate?	Very much—Enough—A little—Not at all
2–Have you experienced a higher-than-usual level of tension or agitation during BJJ practice?	Very much—Enough—A little—Not at all
3–After training, have you felt mentally fatigued to the point of needing more time than usual to recover?	Very much—Enough—A little—Not at all
4–During the week, have you noticed a decrease in motivation or greater difficulty in maintaining your commitment to BJJ training?	Very much—Enough—A little—Not at all
5–Has everyday life stress seemed to interfere with the quality or effectiveness of your BJJ training?	Very much—Enough—A little—Not at all
6–During BJJ, have you found it more difficult to manage frustration, mistakes, or grappling situations perceived as particularly demanding?	Very much—Enough – A little—Not at all

All tests were carried out under the same environmental conditions and at the same times of day, in order to reduce the influence of external factors. Before performing the tests, participants received a brief explanatory briefing intended to clarify the protocol and ensure that execution remained consistent across the different sessions. All participants provided informed consent prior to inclusion in the study. The study was conducted in accordance with the principles of the Declaration of Helsinki. The present study forms part of the research project entitled “Psychophysical perception and body awareness in school and sports contexts through observational and non-invasive tools”, approved by the Ethics Committee of Pegaso University (Prot./E 004726, 15/07/2025).

### 2.2 Statistical analysis

Statistical analysis was organized taking into account the nature of the data collected in the two assessment phases, pre and post intervention, distinguishing between quantitative measures and qualitative evaluations. For the continuous variables, namely the RFD indices obtained through IMTP and the reaction times recorded with the Blaze Pod system, the data distribution was first verified using the Shapiro Wilk test. Variables that met the required assumptions were analyzed with a mixed 2 × 2 ANOVA, which included one within-subject factor (time: pre vs. post) and one between-subjects factor (group: experimental vs. control). In cases where the data did not meet normality requirements, the analysis was conducted using the corresponding non-parametric tests: the Wilcoxon test for pre–post comparisons within the same group and the Mann-Whitney U test for comparisons between the two groups. For each analysis, effect size was also calculated, expressed as partial eta squared (η*p*^2^) in the ANOVA models and as r for the non-parametric tests, following the interpretative thresholds commonly used in the literature. The ordinal data derived from the Likert questionnaire on distress perception was treated as ordinal variables. For each athlete, an overall score was calculated from the mean of the six items, after verifying the internal reliability of the instrument using Cronbach's α coefficient. Since this type of score often shows a distribution that is not fully continuous, the pre–post comparison within the groups was carried out by means of the Wilcoxon test, while the comparison between the experimental and control groups was conducted using the Mann-Whitney U test. Effect size was again quantified through the r coefficient, calculated from the normalized *Z* value. In all statistical procedures, the significance level was set at *p* < 0.05. In addition to significance values, the practical relevance of the results was also considered, jointly interpreting the effect sizes. For the main between-group differences and across assessment time points, 95% confidence intervals (95% CIs) were also calculated in order to quantify the precision of the observed effect estimates and support their applied interpretation beyond statistical significance alone. Data analyses were conducted by a researcher not involved in the delivery of the intervention and blinded to participants' allocation to the experimental and control groups. All analyses were conducted using the statistical software JASP (version 0.95.4).

## 3 Results

**Figure 1:**
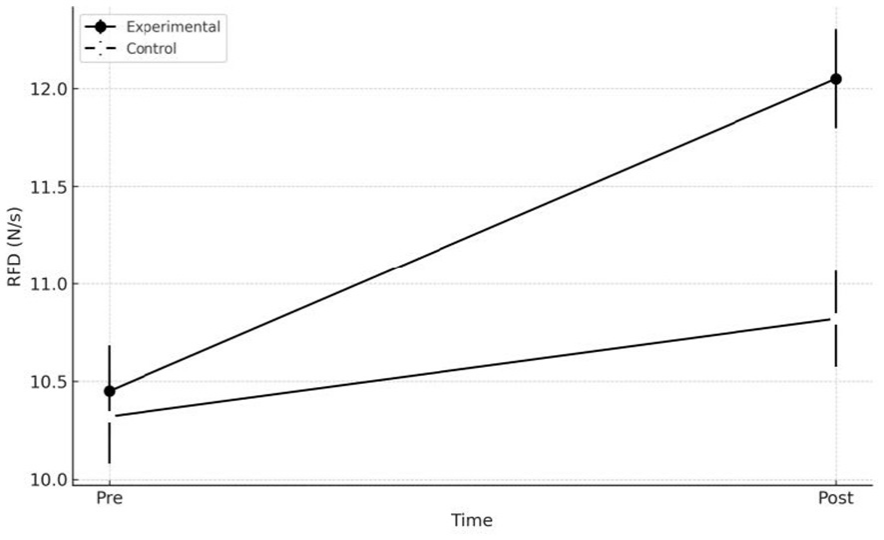
RFD, between-Group Comparison Pre/Post. RFD is expressed in Newtons per second (N/s), a unit that indicates the rate at which force is developed during muscular contraction; higher values represent greater explosive capability. The figure illustrates the changes in mean values in both groups, highlighting the more pronounced increase observed in the experimental group compared with the control group.

At the beginning of the study, the two groups presented comparable values for all variables considered. Neither the RFD indices, nor the Blaze Pod reaction times, nor the scores related to perceived distress showed significant differences, suggesting good initial homogeneity of the sample. With regard to peak RFD, the mixed 2 × 2 ANOVA revealed a significant main effect of time, [*F*_(1.58)_ = 9.84, *p* = 0.003, η*p*^2^ = 0.145], indicating an overall improvement from pre to post intervention. The interaction between time and group was also significant, [*F*_(1.58)_ = 5.72, *p* = 0.020, η*p*^2^ = 0.090], indicating that the progression over time differed between the experimental and control groups. In detail, the experimental group showed a substantial increase, rising from 10.450 ± 1.280 N/s to 12.050 ± 1.390 N/s (+ 15%), with IC 95% [11.54; 12.56]. In the control group, by contrast, the improvement was more limited: from 10.320 ± 1.310 N/s to 10.820 ± 1.350 N/s (+5%), with IC 95% [10.33; 11.31]. The estimated mean pre–post change in the experimental group was + 1.60 N/s (95% CI [0.91, 2.29]), whereas in the control group it was + 0.50 N/s (95% CI [−0.15, 1.15]). Simple comparisons confirmed that the change was significant in the experimental group (*p* < 0.001), whereas in the control group it was only marginal (*p* = 0.078), with IC 95% [0.52; 1.94]. All results are shown in Figure 1.

**Figure 2:**
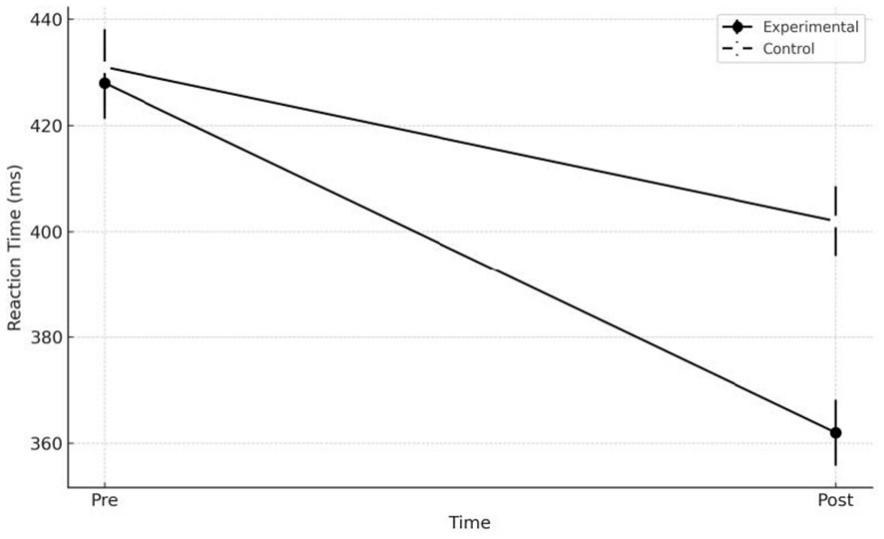
Reaction Time, between-Group Comparison Pre/Post. Comparison between the experimental group and the control group in reaction times measured during the pre- and post-intervention assessments. Reaction time is expressed in milliseconds (ms), a unit that reflects how quickly an athlete responds to a visual stimulus; lower values indicate greater reactivity. The figure shows a reduction in mean reaction times in both groups, with a greater improvement observed in the experimental group.

Reaction time measured through Blaze Pod decreased in both groups, but with a clear advantage for the experimental group. The ANOVA showed a main effect of time, [*F*_(1.58)_ = 18.31, *p* < 0.001, η*p*^2^ = 0.240], and a significant time × group interaction, [*F*_(1.58)_ = 7.95, *p* = 0.007, η*p*^2^ = 0.120]. The experimental group decreased from 428 ± 37 ms at pre-test to 362 ± 34 ms at post-test (−15%), with IC 95% [349.58; 374.42], whereas the control group showed a more modest improvement, from 431 ± 39 ms to 402 ± 36 ms (−7%), with IC 95% [388.85; 415.15]. The estimated mean pre–post reduction in the experimental group was −66 ms (95% CI [−84.36, −47.64]), whereas in the control group it was −29 ms (95% CI [−48.39, −9.61]). Pre–post analyses showed significant differences in both groups, although more pronounced in the EG (*p* < 0.001) compared with the CG (*p* = 0.021), with IC 95% [−58.09; −21.91]. At the end of the intervention, participants in the experimental group were significantly faster in the test compared with the control group (*p* = 0.012). All results are shown in Figure 2.

Table 4 illustrates the trend of RFD resulting from the interaction between the two groups.

**Table 4:** ANOVA – RFD (*N*/*s*).

**Effect**	**df1**	**df2**	***F***	***p***	***ηp^2^***
Time	1	58	9.84	0.003	0.145
Group	1	58	0.46	0.501	0.008
Time × Group	1	58	5.72	0.020	0.090

Table 5 illustrates the trend of reaction times in the two groups.

**Table 5:** ANOVA – reaction time (ms).

**Effect**	**df1**	**df2**	***F***	***p***	***ηp^2^***
Time	1	58	18.31	< 0.001	0.240
Group	1	58	0.36	0.553	0.006
Time × Group	1	58	7.95	0.007	0.120

After the administration of the questionnaire, the results reported in Table 6 were observed for the Control Group.

**Table 6:** Results of the pre/post CG questionnaire responses.

**Questions**	**Response 1**	**Response 2**	**Response 3**	**Response 4**
**1. During BJJ training, have you felt mentally pressured or found it difficult to concentrate?**	**Very**	**Enough**	**A little**	**Not at all**
Frequency pre	4	10	9	7
Percentage pre	13.33	33.3	30.0	23.3
Frequency post	3	9	10	8
Percentage post	10	30	33.3	26.7
**2. Have you experienced a higher-than-usual level of tension or agitation during BJJ practice?**				
Frequency pre	5	9	10	6
Percentage pre	16.7	30	33.3	20
Frequency post	4	10	9	7
Percentage post	13.33	33.33	30	23.3
**3. After training, have you felt mentally fatigued to the point of needing more time than usual to recover?**	**Very**	**Enough**	**A little**	**Not at all**
Frequency pre	3	8	12	7
Percentage pre	10	26.7	40	23.3
Frequency post	3	7	12	8
Percentage post	10	23.3	40	26.7
**4. During the week, have you noticed a decrease in motivation or greater difficulty in maintaining your commitment to BJJ training?**	**Very**	**Enough**	**A little**	**Not at all**
Frequency pre	4	9	11	6
Percentage pre	13.3	30	36.7	20
Frequency post	4	8	11	7
Percentage post	13.3	26.7	36.7	23.3
**5. Has everyday life stress seemed to interfere with the quality or effectiveness of your BJJ training?**	**Very**	**Enough**	**A little**	**Not at all**
Frequency pre	6	10	8	6
Percentage pre	20	33.3	26.7	20
Frequency post	5	9	9	7
Percentage post	16.7	30	30	23.3
**6. During BJJ, have you found it more difficult to manage frustration, mistakes, or grappling situations perceived as particularly demanding?**	**Very**	**Enough**	**A little**	**Not at all**
Frequency pre	5	9	10	6
Percentage pre	16.7	30	33.3	20
Frequency post	4	8	11	7
Percentage post	13.3	26.7	36.7	23.3

In the control group, before the intervention, the responses showed moderate levels of distress. For Question 1, 13.3% of the athletes indicated “Very”, 33.3% “Enough”, while the relative majority chose “A little” 30.0% or “Not at all” 23.3%. For Question 2, the responses were similarly distributed, with 16.7% selecting “Very”, 30.0% “Enough”, 33.3% “A little”, and 20.0% “Not at all”. For Question 3, 10.0% reported high levels, while 40.0% selected “A little”, indicating an intermediate perception of distress; the remaining 26.7% responded “Enough” and 23.3% “Not at all”. Question 4 showed comparable values, with 13.3% selecting “Very”, 30.0% “Enough”, 36.7% “A little”, and 20.0% “Not at all”. For Question 5, 20.0% answered “Very”, 33.3% “Enough”, while the remaining 46.7% indicated “A little” or “Not at all”. For Question 6, the percentages were distributed as follows: 16.7% “Very”, 30.0% “Enough”, 33.3% “A little”, and 20.0% “Not at all”. Overall, the pre-intervention profile of the control group suggests moderate levels of distress, with a slight tendency toward “A little” responses. After the 8 weeks of traditional training, the distribution of responses in the control group remained almost unchanged, confirming the absence of a significant decrease in distress. For Question 1, 10.0% answered “Very”, 30.0% “Enough”, while “A little” and “Not at all” accounted for 33.3% and 26.7% respectively. For Question 2, 13.3% chose “Very”, 33.3% “Enough”, while the remaining 53.3% were distributed between “A little” 30% and “Not at all” 23.3%. For Question 3, 10.0% reported high levels, 23.3% “Enough”, while the majority 40.0% indicated “A little” and 26.7% “Not at all”. For Question 4, 13.3% selected “Very”, 26.7% “Enough”, 36.7% “A little”, and 23.3% “Not at all”. Question 5 showed a slight variation, with 16.7% choosing “Very”, 30.0% “Enough”, and the remainder distributed between “A little” and “Not at all”. Finally, for Question 6, 13.3% reported “Very”, 26.7% “Enough”, while 36.7% answered “A little” and 23.3% “Not at all”. In summary, no relevant changes emerged between pre and post in the control group. After the administration of the questionnaire, the results reported in Table 7 were observed for the Experimental Group.

**Table 7:** Results of the pre/post EG questionnaire responses.

**Questions**	**Response 1**	**Response 2**	**Response 3**	**Response 4**
**1. During BJJ training, have you felt mentally pressured or found it difficult to concentrate?**	**Very**	**Enough**	**A little**	**Not at all**
Frequency pre	6	12	8	4
Percentage pre	20	40	26.7	13.3
Frequency post	2	6	12	10
Percentage post	6.7	20	40	33.3
**2. Have you experienced a higher-than-usual level of tension or agitation during BJJ practice?**				
Frequency pre	7	11	8	4
Percentage pre	23.3	36.7	26.7	13.3
Frequency post	2	7	11	10
Percentage post	6.7	23.3	36.7	33.3
**3. After training, have you felt mentally fatigued to the point of needing more time than usual to recover?**	**Very**	**Enough**	**A little**	**Not at all**
Frequency pre	5	10	11	4
Percentage pre	16.7	33.3	36.7	13.3
Frequency post	1	6	13	10
Percentage post	3.3	20	43.3	33.3
**4. During the week, have you noticed a decrease in motivation or greater difficulty in maintaining your commitment to BJJ training?**	**Very**	**Enough**	**A little**	**Not at all**
Frequency pre	6	12	8	4
Percentage pre	20	40	26.7	13.3
Frequency post	2	6	12	10
Percentage post	6.7	20	40	33.3
**5. Has everyday life stress seemed to interfere with the quality or effectiveness of your BJJ training?**	**Very**	**Enough**	**A little**	**Not at all**
Frequency pre	7	11	8	4
Percentage pre	23.3	36.7	26.7	13.3
Frequency post	2	7	11	10
Percentage post	6.7	23.3	36.7	33.3
**6. During BJJ, have you found it more difficult to manage frustration, mistakes, or grappling situations perceived as particularly demanding?**	**Very**	**Enough**	**A little**	**Not at all**
Frequency pre	6	12	8	4
Percentage pre	20	40	26.7	13.3
Frequency post	1	6	13	10
Percentage post	3.3	20	43.3	33.3

Before the intervention, the experimental group showed slightly higher levels of distress compared with the control group, with a greater concentration of responses in the “Very” and “Enough” categories. For Question 1, 20% of the athletes selected “Very” and 40% “Enough”, while 26.7% indicated “A little” and 13.3% “Not at all”. For Question 2, the responses were similarly distributed: 23.3% answered “Very”, 36.7% “Enough”, while the remaining 40% were divided between “A little” 26.7% and “Not at all” 13.3%. For Question 3, 16.7% reported high levels “Very”, 33.3% indicated “Enough”, and 50% were distributed between “A little” 36.7% and “Not at all” 13.3%. Question 4 showed a similar pattern, with 20% responding “Very”, 40% “Enough”, 26.7% “A little”, and 13.3% “Not at all”. For Question 5, 23.3% answered “Very”, 36.7% “Enough”, while 40% reported lower levels (“A little” 26.7%; “Not at all” 13.3%). Finally, for Question 6, 20% of the athletes indicated “Very”, 40% “Enough”, while the lower categories represented 26.7% “A little” and 13.3% “Not at all”. After the 8 weeks of training based on ecological dynamical constraints, the experimental group showed a clear improvement in distress perception, with a marked redistribution of responses toward the lower categories. For Question 1, only 6.7% indicated “Very”, while “Enough” decreased to 20%. Most responses shifted to “A little” (40%) and “Not at all” 33.3%. For Question 2, 6.7% reported “Very”, 23.3% “Enough”, while 36.7% indicated “A little” and 33.3% “Not at all”. For Question 3, only 3.3% answered “Very”, while “Enough” decreased to 20%; most responses concentrated on “A little” 43.3% and “Not at all” 33.3%. For Question 4, “Very” 6.7% and “Enough” 20% were clearly lower than in the pre-test, while “A little” 40% and “Not at all” 33.3% became predominant. For Question 5, the values followed the same trend: 6.7% indicated “Very”, 23.3% “Enough”, and the sum of “A little” and “Not at all” reached 76.6%. Finally, for Question 6, only 3.3% reported “Very”, 20% “Enough”, while “A little” and “Not at all” together accounted for approximately 76.6% of responses. Overall, the experimental group showed a clear and coherent pattern of distress reduction, with a marked decrease in responses in the higher categories and a consistent increase in the “A little” and “Not at all” categories. The distress questionnaire showed a good level of internal reliability (Cronbach's α = 0.81). Since the aggregated scores did not follow a fully normal distribution, non-parametric tests were used. At baseline, the values of the two groups were comparable [EG: median 2.7 (2.4–3.0); CG: 2.8 (2.5–3.0)], and the Mann-Whitney U test did not reveal differences (*U* = 424.5, *p* = 0.56). At the end of the intervention, the experimental group showed a clear decrease in perceived distress, with a median value of 1.9 [1.6–2.2], and the pre–post comparison showed a significant difference [*Z* = −4.10, *p* < 0.001, *r* = 0.53), with IC 95% (0.21; 0.75)], consistent with a meaningful change rather than one that is solely statistically significant. In the control group, the scores remained substantially unchanged [post: 2.6 (2.3–2.9); Z = −1.21, *p* = 0.226, *r* = 0.16]. The comparison between the groups after the intervention confirmed a significant difference in favor of the experimental group (*U* = 320.0, *p* = 0.004, *r* = 0.38), suggesting that the ecological dynamical constraint based protocol was associated not only with an improvement in performance variables but also with a more marked reduction in perceived distress.

## 4 Discussion

**Figure 3:**
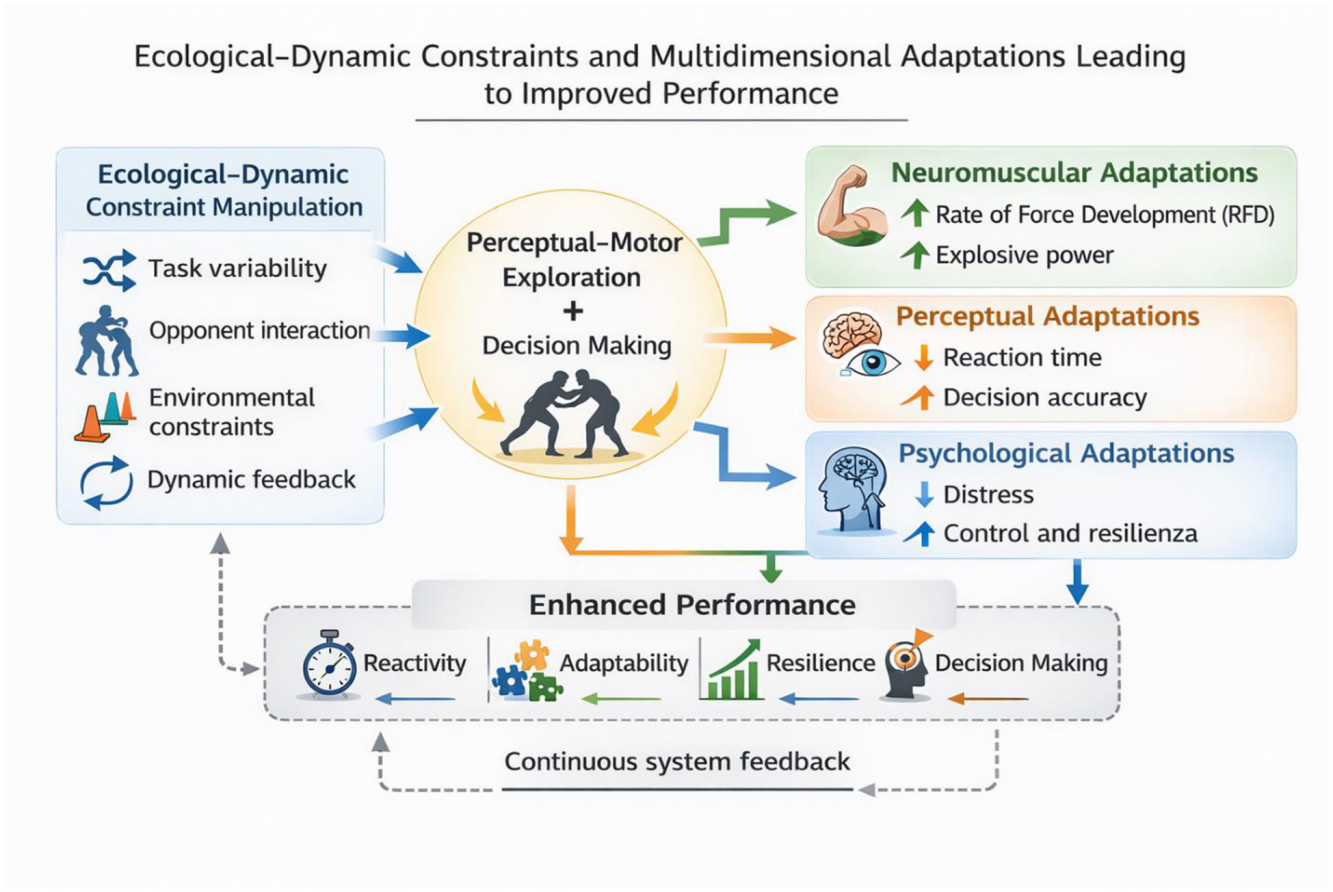
Conceptual framework linking ecological–dynamic constraint manipulation to neuromuscular, perceptual, and psychological adaptations leading to enhanced performance.

The results of the study indicate that the adoption of an approach grounded in the principles of movement ecology and constraint dynamics is associated with more pronounced effects, compared with traditional methodologies, on three fundamental aspects of performance in Brazilian Jiu Jitsu: the neuromuscular ability to generate force rapidly (RFD), reaction speed to visual stimuli, and the subjective perception of distress related to training (Moy et al., 2024). The substantial increase in peak RFD in the experimental group, clearly superior to that observed in the control group, indicates that a practice environment characterized by constraints, variability, and complex perceptual–motor demands may facilitate more effective neuromuscular adaptations than prescriptive technical repetition. In the experimental group, the rise from 10.450 ± 1.280 N/s to 12.050 ± 1.390 N/s resulted in a 15% increase, supported by a significant time × group interaction [(*F*_(1.58)_ = 5.72; *p* = 0.020)]. In the control group, instead, the increase from 10.320 ± 1.310 N/s to 10.820 ± 1.350 N/s resulted in a more modest improvement (+ 5%), which was not statistically significant in the pre–post comparison (*p* = 0.078). This gap between the two groups is relevant and suggest a greater sensitivity of RFD to exposure to variable, information-rich tasks, such as those typical of the ecological–dynamical approach. In line with the initial hypotheses, visuomotor reactivity also showed a markedly greater improvement in the experimental group. The reduction in reaction time from 428 ± 37 ms to 362 ± 34 ms (−15%) represents an important change in terms of operational readiness, supported by a strong main effect of time [(*F*_(1.58)_ = 18.31; *p* < 0.001)] and by a significant interaction (F_(1, 58)_ = 7.95; *p* =0.007). The control group showed a less pronounced improvement, decreasing from 431 ± 39 ms to 402 ± 36 ms (−7%), still significant (*p* = 0.021) but less relevant than that observed in the EG. The direct comparison between groups in the post-test further highlighted the superiority of the experimental intervention (*p* = 0.012). A particularly interesting finding concerns the subjective perception of distress. Only the experimental group showed a consistent and statistically significant decrease in scores, dropping from a median of 2.7 [2.4–3.0] to 1.9 [1.6–2.2], with a substantial effect (*Z* = −4.10, *p* < 0.001, *r* = 0.53). In the control group, by contrast, values remained almost unchanged [pre: 2.8 (2.5–3.0); post: 2.6 (2.3–2.9)], with a non-significant pre–post comparison (*p* = 0.226). The difference between groups in the post-test (*U* = 320.0; *p* = 0.004) shows that a context manipulated through constraints and variability not only promotes neuromuscular and perceptual adaptations but is associated with a reduction in athletes perceived psychological load (Chow et al., 2023). From an applied perspective, the observed effect sizes and the consistent direction of the confidence intervals support the practical relevance of the intervention. In particular, for the main performance outcomes (peak RFD and reaction time), the 95% CIs of the between-group differences at post-test do not include zero, indicating a robust advantage that is likely meaningful also in training practice (Hopkins et al., 2009). Similarly, for distress, the large effect observed in the experimental group (with 95% CIs remaining within the positive range) suggests a non-negligible improvement in perceived stress management within the training context (Kellmann et al., 2018). This result is consistent with the growing body of evidence suggesting that ecological dynamical approaches, thanks to the greater autonomy granted to athletes, the exploratory nature of the task, and the reduced emphasis on prescriptive execution accuracy imposed by the coach, reduce cognitive pressure and promote a greater tolerance for uncertainty (Aliberti et al., 2024). It is important to emphasize that the distress considered in this study refers to the perception of psychological pressure and difficulties in managing the situational demands of training, rather than to perceived physical exertion, anticipatory anxiety states, or simple cognitive fatigue (Hamlin et al., 2019). This combination may foster a sense of self-efficacy, in solving emerging motor problems, thereby contributing to the mitigation of distress related to practice (Di Domenico et al., 2023). Support for the effectiveness of ecological dynamical approaches also emerge from numerous studies conducted in different sports, which collectively confirm that constraint manipulation, task variability, and perceptual–motor exploration are particularly effective levers for promoting functional adaptations. In team sports such as football, as well as in technical and combat sports, training protocols based on representative tasks and variable situations have demonstrated improvements in reactivity, movement quality, and situational adaptability (Ceruso et al., 2025a,b; Giardullo et al., 2025; Esposito et al., 2024a,b; Altavilla et al., 2022). Similar evidence has also emerged in disciplines requiring high levels of coordination and rapid decision-making, suggesting that the principles of the ecological dynamics approach can be effectively applied across different sporting domains (D'Isanto et al., 2025; Esposito et al., 2025a,b,c). This convergence of results, observed in sporting contexts other than Brazilian Jiu-Jitsu, further strengthens the interpretation of the present study's findings, suggesting that the ecological dynamics approach may represent a promising and potentially versatile methodological framework, capable of generating benefits that transcend the specificity of the discipline. A conceptual representation of the proposed relationships between ecological–dynamic constraint manipulation, perceptual–motor processes, and multidimensional adaptations is presented in Figure 3.

Although the observed improvements are consistent with the theoretical principles of ecological dynamics, the underlying neural and psychophysiological mechanisms were not directly measured in the present study. Therefore, interpretations concerning possible adaptations in motor control processes, autonomic regulation, or cognitive management should be regarded as plausible explanations supported by literature, rather than as demonstrated causal evidence.

### 4.1 Practical applications

From an applied perspective, these results suggest that integrating elements of the ecological approach into BJJ training programs improves reactivity to opponent stimuli, promotes specific neuromuscular adaptations, reduces the perception of psychological stress, and increases the overall quality of motor learning. For coaches, therefore, introducing task constraints, situational variability, and dynamic grappling scenarios may represent a useful methodological development in support of performance.

### 4.2 Limits and future prospects

One limitation of the study concerns the characteristics of the sample and the consequent generalizability of the findings. Participants were recruited through convenience sampling within a specific training context, a condition that restricts the extension of the results to other sporting environments or athlete populations. However, this methodological choice allowed for the maintenance of homogeneous training conditions, limiting the influence of contextual variables such as differences in programmes, workload, and coaching style, which in combat sports may significantly affect training-induced adaptations. Consequently, the findings should be interpreted as primarily transferable to practice contexts characterized by similar levels of experience and training structure, rather than as representative of the entire population of Brazilian Jiu-Jitsu practitioners. In this sense, the study offers analytical and applied generalizability, providing useful indications for practice and for the design of future research involving larger and more diverse samples. Another limitation relates to the use of an *ad hoc* questionnaire to assess distress, which does not allow direct comparison with fully validated psychometric scales widely used in the sport psychology literature. This reduces comparability with previous studies that have employed standardized instruments to measure stress and psychological load. However, in line with previous sport research adopting context-specific tools to capture discipline-specific perceptions, this choice was guided by the need to identify situational forms of distress characteristic of Brazilian Jiu-Jitsu, which are difficult to detect using general-purpose questionnaires. The good internal consistency observed suggests an acceptable level of reliability; nevertheless, future research should include systematic psychometric validation procedures, such as content validity analyses involving field experts, factor analyses to define the construct structure, and assessments of convergent validity with standardized distress and sport stress instruments. Furthermore, distress was assessed exclusively through a subjective measure, without integration with objective psychophysiological indicators such as heart rate variability (HRV), electrodermal response, or endocrine markers such as salivary cortisol. The absence of triangulation between perceptual dimensions and physiological responses represents a limitation, as it does not allow full differentiation between perceived psychological adaptations and modifications in the underlying neuroendocrine or autonomic systems. Future research should integrate subjective and physiological measures to further investigate the relationship between neuromuscular adaptations, autonomic regulation, and perceived psychophysical load in combat sports. An additional limitation concerns the potential influence of confounding factors that were not fully controlled. Variables such as individual differences in technical experience level, training history, motivation, recovery strategies outside training, and daily stress may have contributed, at least in part, to the observed adaptations. Although the study sought to minimize these influences by maintaining homogeneous practice conditions and structured protocols, the applied nature of the sporting context does not permit complete control of all potentially relevant variables. Consequently, the findings should be interpreted with these possible influences in mind, and future research should include additional control measures to more precisely isolate the effects of the intervention. The results are primarily transferable to athletes with an intermediate or advanced amateur level of experience engaged in structured training contexts similar to the one examined. Their extension to elite athletes or beginners requires caution, as differing performance demands and learning trajectories may modulate responses to constraint-based interventions in different ways. For related combat or grappling disciplines, the principles of the ecological dynamics approach appear potentially transferable; however, regulatory and technical–tactical differences necessitate sport-specific empirical verification.

## 5 Conclusion

Overall, the results of this study show that an approach inspired by ecological–dynamical principles may represent an effective methodological proposal for Brazilian Jiu Jitsu. The athletes who followed the protocol based on constraint manipulation and situational variability demonstrated more substantial improvements both in their ability to generate force rapidly and in their speed of response to visual stimuli, compared with practitioners trained through traditional methods. These changes were also accompanied by a significant reduction in perceived distress, indicating that a more dynamic practice environment centered on exploration may have a positive effect not only on performance but also on the athletes' psychological experience. At the same time, the study shows how attention to psychological aspects, often overlooked in technical training, can offer a more complete understanding of the athlete's adaptation processes. While acknowledging certain limitations, including the use of a non-validated questionnaire designed specifically to capture forms of distress typical of BJJ, the work provides useful indications for practice and opens interesting perspectives for future research. It will indeed be important to verify the stability of the effects over the long term and to assess the extent to which the ecological approach may influence tactical and decision-making aspects linked to the complexity of combat. In summary, the data collects highlight that introducing elements of the ecological approach into Brazilian Jiu Jitsu training not only improves performance quality but also promotes a broader form of adaptation that encompasses the athlete's neuromuscular, perceptual, and psychological dimensions. This methodological orientation may therefore represent a promising direction for the evolution of training in grappling disciplines.

## Data Availability

The raw data supporting the conclusions of this article will be made available by the authors, without undue reservation.
